# Progress in modelling ATP13A2-linked neurodegeneration

**DOI:** 10.1038/s41531-026-01331-w

**Published:** 2026-04-04

**Authors:** Benedetta Balbo, Rémi Kinet, Laura Civiero, Benjamin Dehay

**Affiliations:** 1 Department of Biology, University of Padova, Padova, Italy; 2 Univ. Bordeaux, CNRS, IMN, UMR 5293, F-33000 Bordeaux, France; 3 IRCCS San Camillo Hospital, Venice, Italy

**Keywords:** Cell biology, Diseases, Genetics, Neurology, Neuroscience

## Abstract

ATP13A2 is a lysosomal P5-ATPase highly expressed in the central nervous system, regulating polyamine, metal cation, and calcium homeostasis. Loss-of-function mutations cause an autosomal recessive juvenile form of Parkinson’s disease called Kufor-Rakeb syndrome and other neurodegenerative disorders. Since the first clinical discovery of the Kufor-Rakeb syndrome, numerous ATP13A2-related models have emerged, leading to significant advances in understanding the physiology and pathophysiology of this protein. This review summarizes ATP13A2 structure, function, pathology, and insights gained from cellular and animal models, highlighting their value for elucidating disease mechanisms and therapeutic development across species and experimental systems, relevant to neurodegeneration research broadly.

## Introduction

**Fig. 1:**
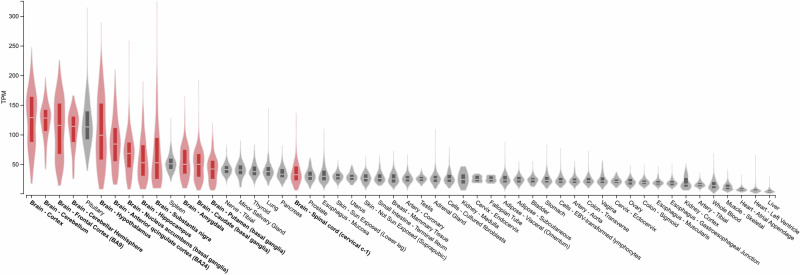
Bulk tissue gene expression for ATP13A2. Data are expressed as transcripts per million (TPM) in different tissue types and are sorted in descending order, with central nervous system-related regions represented in red and others in gray. Data adapted from the GTEx Portal.

**Fig. 2:**
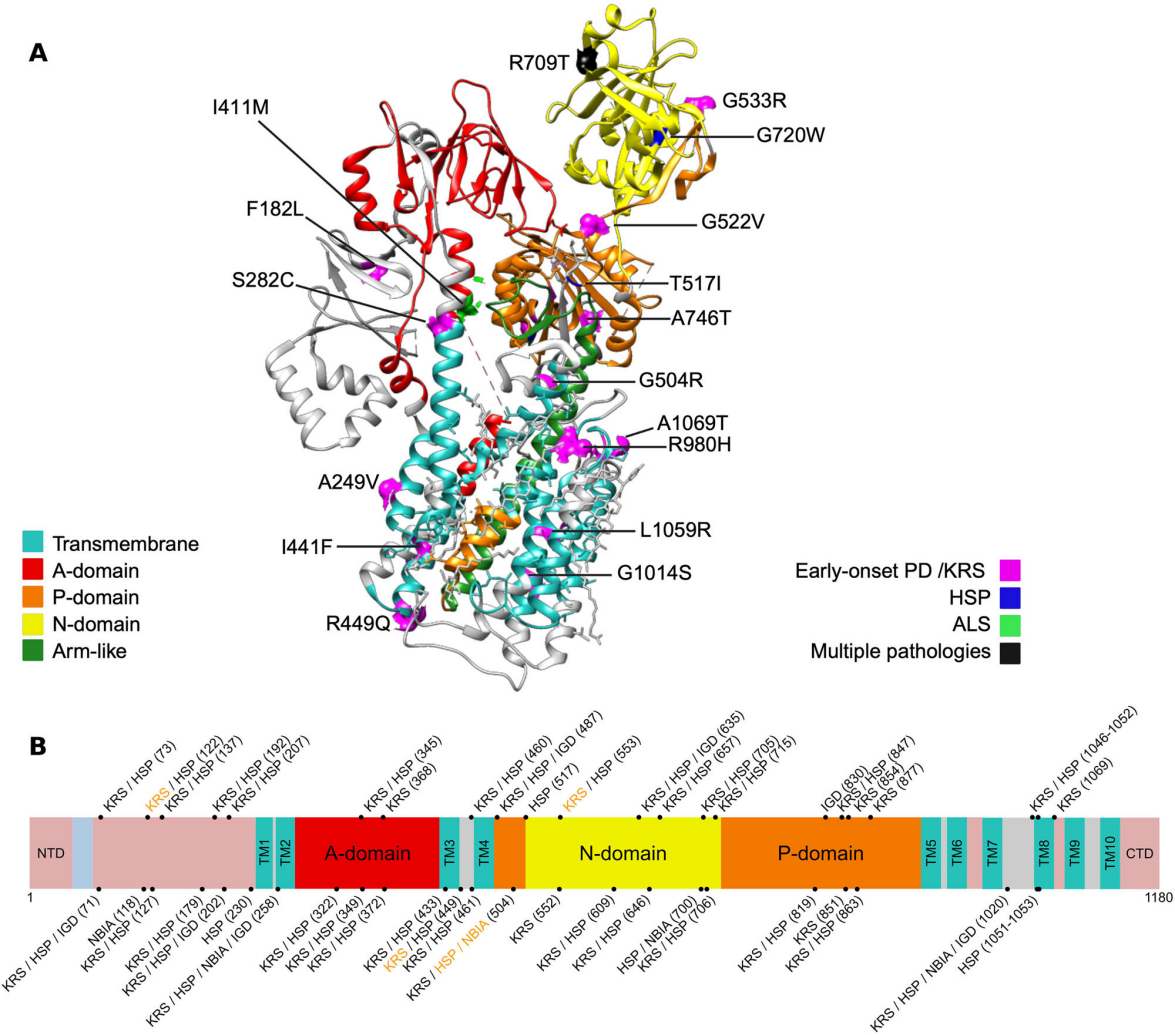
Architecture of human ATP13A2. **A** Schematic representation of domain organization and distribution of reported variants along the protein, annotated according to their associated clinical phenotypes. Variants are shown above the schematic, and black lines indicate previously identified variants in ATP13A2-related pathologies. **B** Topology diagram of hATP13A2. Transmembrane helices TM1–TM10 are numbered. TM transmembrane domain, A-domain actuator domain, P-domain phosphorylation domain, N-domain nucleotide binding domain, NTD N-terminal extension domain, CTD C-terminal extension domain. The same domain abbreviations are used in all figures.

ATP13A2 is a transmembrane lysosomal P5-ATPase involved in the transport of polyamines and is important in the regulation of basal ganglia functions^1^, divalent heavy metal cations^2^, and Ca^2+^^3^. Indeed, ATP13A2 is largely expressed in the brain, mainly in cortical and cerebellar regions, but also in the hypothalamus and the basal ganglia nuclei (Fig. 1). ATP13A2 loss of function is the cause of an autosomal recessive juvenile-onset form of Parkinson’s disease (PD) called Kufor-Rakeb syndrome (KRS)^4–7^, but has also been associated with neuronal ceroid lipofuscinosis (NCL)^8^, hereditary spastic paraplegia (HSP)^9,10^, and amyotrophic lateral sclerosis (ALS)^11,12^. To date, approximately fifty KRS patients have been identified, spread across the Middle East, South America, Asia, and Europe. NIH ClinVar lists 41 pathogenic *ATP13A2* mutations linked to KRS, including nonsense, frameshift, and splice-variant mutations (Table 1). Although pathogenic mutations can be located in different regions of *ATP13A2* (P5-ATPase, E1-E2 ATPase, Hydrolase regions), there is no clear characteristic segregation of the affected regions in the final phenotype between KRS, HSP, and ALS^9^ (Fig. 2**)**. First described in 1994 in a consanguineous family from Jordan^13^, KRS is characterized by a juvenile-onset with symptoms appearing mainly during adolescence, but can appear as early as one year old^14^. Although heterogeneous, symptoms include pyramidal and extra-pyramidal signs, myoclonus, supranuclear gaze palsy, dementia, and other neuropsychiatric symptoms^4,7,13,15,16^. Interestingly, from a treatment perspective, most KRS patients respond to levodopa treatment. At an anatomopathological level, patients demonstrate cerebellar and cortical atrophy, especially in the frontoparietal areas^6,17^, and reduced DAT-scan signal in the putamen^6^. To our knowledge, the only post-mortem neuropathological KRS description reported by Chien et al.^6^ shows the absence of α-synuclein (α-syn)-positive, tau-positive, β-amyloid-positive, and TDP-43-positive protein accumulation. However, a severe loss of dopaminergic neurons in the substantia nigra (SN), accompanied by the presence of lipofuscin in neuronal and glial cells, has been confirmed, as well as the detection of iron deposit accumulation^7^. Nonetheless, heterozygous *ATP13A2* mutations have been reported as a potential risk factor for early-onset PD^18,19^, but not for late-onset PD^20^, reinforcing the importance of the autophagy-lysosomal pathway (ALP) in PD pathogenesis. The rarity of this disease, as well as the lack of analysis of patient samples, means that many aspects, including the pathological mechanisms underlying ATP13A2 loss-of-function in the pathogenesis of KRS and dopaminergic neurodegeneration in patients, remain unknown.

**Table 1:** Summary of pathogenic mutations in *ATP13A2* associated with KRS

Name	Protein change	Conditions(s)	Accession	Variant type	Molecular consequence
NM_022089.4(ATP13A2):c.2539C>T (p.Gln847Ter)	Q803*, Q842*, Q847*	SPG78|KRS	VCV003755807	single nucleotide variant	nonsense
NM_022089.4(ATP13A2):c.2366_2367del (p.Leu789fs)	L784fs, L789fs	SPG78|KRS	VCV003762968	Microsatellite	frameshift variant
NM_022089.4(ATP13A2):c.2113C>T (p.Gln705Ter)	Q700*, Q705*	SPG78|KRS	VCV001996186	single nucleotide variant	nonsense
NM_022089.4(ATP13A2):c.1970del (p.Pro657fs)	P652fs, P657fs	SPG78|KRS	VCV003757378	Deletion	frameshift variant
NM_022089.4(ATP13A2):c.1932del (p.Ala646fs)	A641fs, A646fs	SPG78|KRS	VCV002923611	Deletion	frameshift variant
NM_022089.4(ATP13A2):c.1825G>T (p.Glu609Ter)	E604*, E609*	SPG78|KRS	VCV001999337	single nucleotide variant	nonsense
NM_022089.4(ATP13A2):c.1296dup (p.Ser433fs)	S433fs, S428fs	SPG78|KRS	VCV002013159	Duplication	frameshift variant
NM_022089.4(ATP13A2):c.619C>T (p.Gln207Ter)	Q202*, Q207*	SPG78|KRS	VCV001442231	single nucleotide variant	nonsense
NM_022089.4(ATP13A2):c.217del (p.Val73fs)	V73fs	SPG78|KRS	VCV002931003	Deletion	frameshift variant
NM_022089.4(ATP13A2):c.1033_1034del (p.Leu345fs)	L340fs, L345fs	SPG78|KRS	VCV002629777	Microsatellite	frameshift variant
NM_022089.4(ATP13A2):c.1459C>T (p.Arg487Ter)	R487*, R482*	SPG78|KRS|IGD	VCV000520750	single nucleotide variant	nonsense
NM_022089.4(ATP13A2):c.604del (p.His202fs)	H202fs, H197fs	SPG78|KRS|IGD	VCV001751269	Deletion	frameshift variant
NM_022089.4(ATP13A2):c.3205del (p.Ala1069fs)	A1025fs, A1064fs, A1069fs	KRS	VCV003897662	Deletion	frameshift variant
NM_022089.4(ATP13A2):c.2629G>A (p.Gly877Arg)	G877R, G833R, G872R	KRS	VCV000066099	single nucleotide variant	missense variant
NM_022089.4(ATP13A2):c.2561T>G (p.Met854Arg)	M810R, M854R, M849R	KRS	VCV000066098	single nucleotide variant	missense variant
NM_022089.4(ATP13A2):c.2552_2553del (p.Phe851fs)	F807fs, F846fs, F851fs	KRS	VCV000030834	Deletion	frameshift variant
NM_022089.4(ATP13A2):c.2540_2550del (p.Gln847fs)	Q803fs, Q842fs, Q847fs	KRS	VCV002572413	Deletion	frameshift variant
NM_022089.4(ATP13A2):c.1749+442_2251+512del		KRS	VCV000978042	Deletion	splice acceptor variant|splice donor variant
NM_022089.4(ATP13A2):c.1633_1654dup (p.Leu552fs)	L547fs, L552fs	KRS	VCV000001220	Duplication	frameshift variant
NM_022089.4(ATP13A2):c.1101_1102dup (p.Thr368fs)	T363fs, T368fs	KRS	VCV000030833	Microsatellite	frameshift variant
NM_022089.4(ATP13A2):c.3157_3158del (p.Leu1053fs)	L1009fs, L1053fs, L1048fs	KRS|SPG78	VCV000660925	Microsatellite	frameshift variant
NM_022089.4(ATP13A2):c.3153dup (p.Ser1052fs)	S1052fs, S1008fs, S1047fs	KRS|SPG78	VCV001968613	Duplication	frameshift variant
NM_022089.4(ATP13A2):c.3136G>T (p.Glu1046Ter)	E1002*, E1041*, E1046*	KRS|SPG78	VCV001075283	single nucleotide variant	nonsense
NM_022089.4(ATP13A2):c.2587del (p.Val863fs)	V863fs, V858fs, V819fs	KRS|SPG78	VCV002022009	Deletion	frameshift variant
NC_000001.11:g.(?_16991714)_(16992601_?)del		KRS|SPG78	VCV000660676	Deletion	
NM_022089.4(ATP13A2):c.2146del (p.Asp715_Leu716insTer)		KRS|SPG78	VCV001075284	Deletion	nonsense
NM_022089.4(ATP13A2):c.1382del (p.Ala461fs)	A456fs, A461fs	KRS|SPG78	VCV003763361	Deletion	frameshift variant
NM_022089.4(ATP13A2):c.1378del (p.Arg460fs)	R455fs, R460fs	KRS|SPG78	VCV002179757	Deletion	frameshift variant
NM_022089.4(ATP13A2):c.1113del (p.His372fs)	H372fs, H367fs	KRS|SPG78	VCV001385899	Deletion	frameshift variant
NM_022089.4(ATP13A2):c.965del (p.Gln322fs)	Q317fs, Q322fs	KRS|SPG78	VCV002021882	Deletion	frameshift variant
NM_022089.4(ATP13A2):c.572dup (p.Arg192fs)	R192fs, R187fs	KRS|SPG78	VCV002039313	Duplication	frameshift variant
NM_022089.4(ATP13A2):c.533_536dup (p.Gln179fs)	Q179fs, Q174fs	KRS|SPG78	VCV002064277	Duplication	frameshift variant
NM_022089.4(ATP13A2):c.409del (p.Val137fs)	V137fs	KRS|SPG78	VCV000859962	Deletion	frameshift variant
NM_022089.4(ATP13A2):c.379del (p.Asp127fs)	D127fs	KRS|SPG78	VCV003748836	Deletion	frameshift variant
NM_022089.4(ATP13A2):c.217dup (p.Val73fs)	V73fs	KRS|SPG78	VCV002151865	Duplication	frameshift variant
NM_022089.4(ATP13A2):c.213G>A (p.Trp71Ter)	W71*	KRS|SPG78	VCV002419737	single nucleotide variant	nonsense
NC_000001.10:g.(?_17330807)_(17332293_?)del		KRS|SPG78	VCV001458906	Deletion	
NC_000001.10:g.(?_17316166)_(17332293_?)del		KRS|SPG78	VCV001456750	Deletion	
NM_022089.4(ATP13A2):c.774G>A (p.Trp258Ter)	W253*, W258*	KRS|SPG78|NBIA	VCV002046647	single nucleotide variant	nonsense
NM_022089.4(ATP13A2):c.2455C>T (p.Arg819Ter)	R819*, R814*	KRS|SPG78	VCV000502116	single nucleotide variant	nonsense|intron variant
NM_022089.4(ATP13A2):c.1903C>T (p.Gln635Ter)	Q635*, Q630*	IGD|SPG78|KRS	VCV000465252	single nucleotide variant	nonsense

NIH ClinVar list of *ATP13A2*-related mutations filter to “pathogenic” mutations, and at least related to the KRS condition. *KRS* Kufor-Rakeb Syndrome, *SPG78* Autosomal recessive spastic paraplegia type 78, IGD Inborn genetic diseases.

**Fig. 3:**
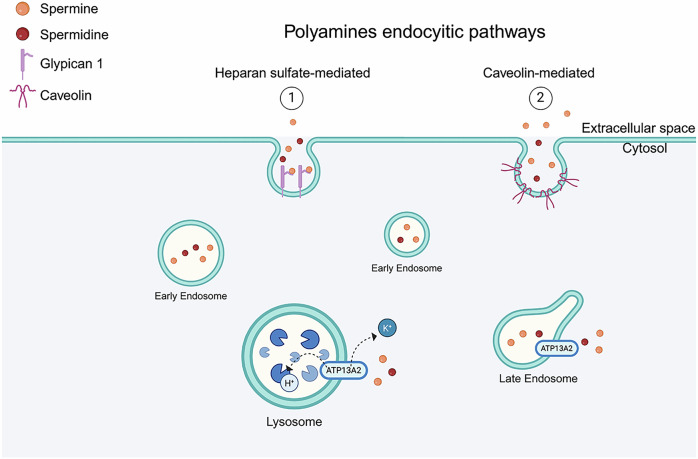
ATP13A2 H^+^/K^+^ and polyamine homeostasis process. Polyamines are either biosynthesized in the cell or taken up through the endocytic pathway. Once internalized, they are released into the cytosol from endosomes through the action of ATP13A2. In addition to its role in polyamine transport, ATP13A2 also functions as an H⁺/K⁺ transporter, extruding K⁺ from lysosomes while importing H⁺, thereby contributing to lysosomal ion homeostasis and acidification.

ATP13A2 comprises 10 transmembrane domains, 3 cytoplasmic domains, namely A- (actuator), N- (nucleotide binding), and P- (phosphorylation) domains, and the C-terminal regulatory domain. Together, these structural elements form two substrate-binding sites and one release site^21–25^. ATP binding to the cytoplasmic domains induces a convergence of the A, N, and P domains, enabling the catalytic action of the enzyme. The P-domain mediates phosphorylation via the DKTG amino acid motif, while the A-domain contributes to dephosphorylation through the TGE sequence. The KGSPE motif participates in the ATP coordination within the N-domain and facilitates structural rearrangements required for catalytic cycling. During ADP release, an outward rotation of the N-domain of approximately 25° occurs, leading to remodeling of the transmembrane architecture and enabling recruitment of a polyamine substrate into the luminal binding pocket Site_1_^21^. This conformation enables the transport of polyamines, with a highest affinity for spermine^26^ and metal cations through the Post-Albers reaction, characterized by a series of intermediate states like previously described, with polyamine recruitment from the lumen side and subsequent release into the cytoplasm^21,24,25^. As a result, the ATP13A2 protein appears to be a key player in cellular polyamine metabolism^26^, heavy metal homeostasis^27–29^, and lysosomal functions, such as regulation of luminal pH and enzymatic activity^30,31^ (Fig. 3).

**Fig. 4:**
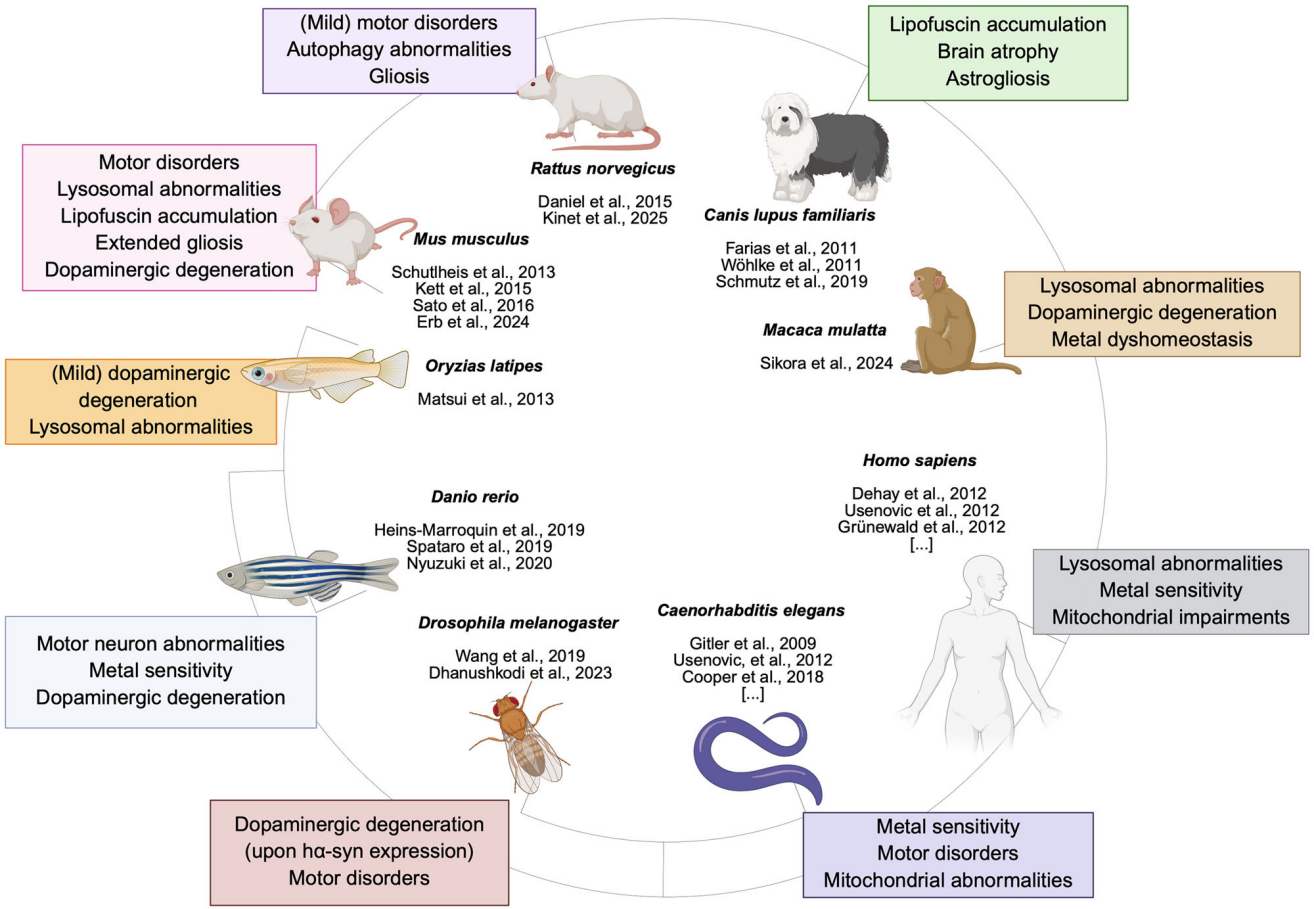
Tree of ATP13A2-associated models. Circular phylogenetic tree generated with iTOL. The tree depicts the animal models used to recapitulate ATP13A2-linked neurodegeneration, along with supporting evidence from patient studies. For each species, the reported phenotypes and pathological features associated with ATP13A2 dysfunction are indicated, and the corresponding references are listed within the figure.

Given the limited number of characterized KRS patients and the scarcity of post-mortem neuropathological descriptions, studying this pathology across diverse experimental models is essential to better understand the role of ATP13A2, to complement clinical and cellular data, and to provide a platform for exploring pathophysiology and testing therapeutic hypotheses. Cellular models allowed significant comprehension of the roles of ATP13A2, including the identification of numerous types of in vitro models, such as fibroblasts derived from ATP13A2-deficient patients carrying the mutations L3292, L6025, or T512I^30,32^, human neuroblastoma *ATP13A2* KO^26,30,31,33–37^, silenced with small-hairpin RNA (shRNA) or small-interfering RNA (siRNA) targeting *ATP13A2*^30,33,36,38,39^, or overexpressing ATP13A2^26,35,37,38,40^. Moreover, the use of induced pluripotent stem cell (iPSC)-derived ATP13A2-related cells has recently been reported^36^. From another perspective, animal models enable an integrated study of the consequences of the loss of the ATP13A2 protein on the entire nervous system by assessing motor, behavioral, or biochemical phenotypes. For example, animal models enable the examination of damage to dopaminergic or motor neurons during development or aging, and to visualize lysosomal abnormalities in a living brain. Other advantages include the possibility of manipulating the gene at different stages (embryonic development or induced in adulthood). Finally, animal models provide a framework for testing innovative therapeutic approaches (gene therapy, chelating drugs, etc.) before any clinical application. Various models have already been crucial in the neurodegeneration field, such as PD linked to the *SNCA*, *LRRK2*^41^, *PRKN*^42^, and *PINK1*^43^ genes. Similarly, for KRS, models offer the possibility of connecting the molecular functions of ATP13A2 to the clinical symptoms observed and identifying pharmacological targets. Herein, we compile and summarize the results of reported ATP13A2-related models in the literature, from immortalized cells to non-human primates (Fig. 4 and Table 2).

**Table 2:** Invertebrate and mammalian models of ATP13A2-related animal models in the literature

Reference	Year	Organism	Model type	Pathological induction	Dopaminergic degeneration	Motor disorders
Gilter et al.	2009	*C. elegans*	- catp-6 deletion strain RB2510 (W08D2.5(ok3473) IV.) - catp-6 expression (dat-1 promoter)	- hα-syn expression (dat-1 promoter)	- catp-6 expression reverses α-syn-induced degenerescence	
Usenovic et al.	2012	*C. elegans*	- catp6 RNAi (dat-1 promoter)	- hα-syn expression (dat-1 promoter)	- No DA neurodegeneration in the absence of α-syn - enhanced α-syn-induced DA neurodegeneration upon the RNAi KD	/
Cooper et al.	2018	*C. elegans*	- catp-6 deletion strain RB2510 (W08D2.5(ok3473) IV.)	- Ubiquitous hα-syn expression (eft-3 promoter) - 4 mM paraquat exposure - 500 mM NaCl exposure	- >20% DA neurodegeneration at 15 days old, but not exacerbated by α-syn expression	- Crawling speed and basal slowing decreased in catp-6 compared to WT
Baesler et al.	2019	*C. elegans*	- catp-6 deletion strain RB2510 (W08D2.5(ok3473) IV.)	- Zinc exposure	/	/
Narayanaswamy et al.	2019	*C. elegans*	- catp-6 deletion strain RB2510 (W08D2.5(ok3473) IV.)	/	/	/
Ugolino et al.	2019	*C. elegans*	- hATP13A2-GFP expression (dat-1 promoter)	- MnCl_2_ exposure	- Rescue of DA neurons integrity by hATP13A2 expression up to 350 mM MnCl_2_ exposure	/
Van Veen et al.	2020	*C. elegans*	- catp-5, or catp-6 or catp-7 deletion	- Polyamine exposure	/	/
Anand et al.	2020	*C. elegans*	- catp-6 KO - catp-6 KO + TFEB enhancer	- Rotenone or ferric ammonium citrate exposure	/	
Vrijsen et al.	2020	*C. elegans*	- catp-6 (ok3473) KO - catp-6 reexpression - catp6 (D465N) catalytically inactive mutant	- Rotenone exposure	/	/
Wang et al.	2019	*D. melanogaster*	- dATP13A2 silencing through UAS-Gal4 RNAi system	/	/	- Reduced climbing ability
Dhanushkodi et al.	2023	*D. melanogaster*	- dATP13A2 silencing through UAS-Gal4 RNAi system	- Expression of WT, A53T, A30P hα-syn	- 50% reduction in the number of TH^+^ neurons only in the PPL2 cluster	- Altered circadian rhythmicity and reduced locomotor activity
Sparato et al.	2019	*D. rerio*	- Splice-blocking morpholinos mediated KD with 2 antisense oligonucleotides targeting exon 4 and exon 6	/	/	/
Heins-Marroquin et al.	2019	*D. rerio*	- Single point mutation in exon induced in adult males treated with ENU	- MnCl_2_ exposure	/	- Movement loss upon MnCl_2_ exposure
Nyuzuki et al.	2020	*D. rerio*	- CRISPR/Cas9 mediated KO through a 10 bp deletion in the exon 2	/		/
Matsui et al.	2013	*O. latipes*	- TILLING-based mutant skipping exon 13	/	- Mild age-dependent loss of TH^+^ neurons	- Increase in swimming duration
Schultheis et al.	2013	*M. musculus*	- Replacement vector strategy deleting exons 12-15	/	-	- Mild motor disorders starting from 23^rd^ month of age
Kett et al.	2015	*M. musculus*	- LoxP sites around exons 2 and 3	/	-	- Mild motor disorders starting from 18^th^ month of age
Sato et al.	2016	*M. musculus*	- LoxP sites around exons 2 and 3 combined with the Nestin-Cre driver system	/	/	- Mild motor disorders starting from 18^th^ month of age
Erb et al.	2024	*M. musculus*	- combined with unilateral injections of AAV vectors into the SNpc	/	- 34.68% loss of TH^+^ neurons in the injected hemisphere	/
Daniel et al.	2015	*R. norvegicus*	- AAV-hATP13A2 - AAV-ATP13A2-D513N mutated	AAV-hsyn SN	- AAV-hATP13A2 is insufficient to rescue TH and Nissl depletion from AAV-hsyn - AAV-ATP13A2-D513N induces TH and Nissl depeletion	- Motor asymmetry deficit with AAV-ATP13A2-D513N
Kinet et al.	2025	*R. norvegicus*	ATP13A2 KO CrisprCas9	- AAV-A53T - AAV-hTyr	No neurodegeneration	- Deficient single pellet reaching 12mo
Farias et al.	2011	*C. lupus familiaris*	Spontaneous mutation	/	/	cerebellar ataxia, tonic–clonic seizures
Wohlke et al.	2011	*C. lupus familiaris*	Spontaneous mutation	/	/	Lack of motor coordination, problems jumping, seizures
Schmutz et al.	2019	*C. lupus familiaris*	Spontaneous mutation	/	/	Impaired ability to navigate stairs and to jump, trembling, seizures, stiffness, weakness, loss of coordination
Sikora et al.	2024	*M. mulatta*	- Supranigral injection of AAV containing shRNA ATP13A2	/	30% loss of TH+ neurons in SN 55% DA depletion in the putamen 30% DA depletion in the caudate nucleus	/

## Non-mammalian models

### Worm Models

Since 2009, multiple articles have used the *Caenorhabditis elegans* (*C. elegans*) model to study the role of the nematode *ATP13A2* paralogous *catp-5*, *catp-6*, and *catp-7*. The catp-6 orthologue has a vesicular and membrane localization in neurons, and is colocalized with early endosomal marker Rab5, making it more suitable for the study of PD compared to catp-5 and catp-7, which are colocalized and expressed to the apical membrane in intestinal and spermathecal cells or plasma membranes of the intestine, hypodermis, and spermatids^44^. The *catp-6* knockdown, combined with the expression of α-syn in *C. elegans*, has been associated with an increase of α-syn misfolding in an age-dependent manner and dopaminergic neurodegeneration^45,46^. The neurodegeneration caused by α-syn is rescued by the co-expression of the ATP13A2 orthologue in *C. elegans*^45^, demonstrating the importance of this lysosomal ATPase in the regulation and toxicity of α-syn levels in the dopaminergic system. Moreover, catp-6 deletion is linked to increased sensitivity to other neurodegenerative processes, such as oxidative stress. Indeed, in α-syn-expressing and catp-6-deficient animals, exposure to molecules known to be pathogenic in PD, such as paraquat^47^ or rotenone^33^ pesticides, zinc^27^, iron^28^, and osmotic stress^47^ leads to a significant decrease in survival rate. Interestingly, and consistent with KRS clinical observations, the catp-6 deletion is sufficient to induce a motor deficit, characterized in *C. elegans* by a significant reduction in the number of body bends per 30 seconds. In addition, when looking at autophagic alterations caused by catp-6 loss of function, a decreased level of LC3-II protein was observed, suggesting a lack of autophagosome production. Moreover, the authors detected a high level of premature forms compared to mature forms of cathepsin D (catD), displaying a lack in the conversion of catD, concomitant with an alkalinized luminal lysosomal pH^28^. Furthermore, a drop in multiple autophagy-related mRNAs was observed (atg-18, lmp-1, vsp-11, sul-2, vha-16, and ctsb), participating in the ALP-defective process in the ATP13A2 deletion condition^28^. These combined findings place ATP13A2 as a key regulator of the ALP, through direct action on the lysosomal luminal pH and enzymatic control, but also on the autophagy flux. At the mitochondrial level, catp-6 deletion affects mitochondrial integrity and function, reducing the maximum mitochondrial oxygen consumption capacity, and significantly increases rotenone sensitivity^28^. Supporting this evidence, the expression of human ATP13A2 in the worm’s dopaminergic neurons leads to a significant increase in resistance to neurodegeneration after acute manganese exposure up to 350 mM^29^. The loss of function in ATP13A2, therefore, leads to significant mitochondrial dysfunction, further contributing to the pathogenesis of PD and KRS. It is important to notice that the *C. elegans* model permitted the description of the lysosomal Ca^2+^ import function of catp-6, reported as the first lysosome-specific Ca^2+^ importer by Narayanaswamy and colleagues^3^. Finally, a later study looked at the *catp-6* role in polyamine export in the *C. elegans* model^26^. This article by Van Venn and colleagues describes the affinity of ATP13A2 with polyamine, especially for spermine and spermidine, and the protective role of ATP13A2 against spermidine toxicity^26^. Indeed, ATP13A2 promotes the cellular polyamine uptake through endocytosis, and its loss of function exacerbates the polyamine toxicity, resulting in significantly smaller worms^26^. ATP13A2-related *C. elegans* models have led to significant advances in understanding the ATP13A2 mechanisms, proving the neurotoxic effect of ATP13A2 deletion and its role in various pathogenic mechanisms, such as mitochondrial alterations, sensitivity to heavy metals and pesticides, exacerbating the harmful mechanisms involved in PD and KRS.

### Fly Models

The fruit fly *Drosophila melanogaster* shares many genes and biological pathways that can be easily perturbed through genetic experiments, providing a powerful platform for investigating neurodegenerative diseases^48,49^. The dopaminergic system is well-characterized and conserved, comprising approximately 127 bona fide dopaminergic neurons^50^ that regulate multiple functions, including circadian rhythms, feeding, learning, and locomotion. Dopaminergic neurons in the Drosophila brain are organized into eight different types, which are classified into three different clusters: PAM (protocerebral anterior medial), PPL1 (protocerebral posterior lateral 1), and PPL2 clusters^50^. PPL1 primarily projects to Drosophila brain areas critical for learning, memory, and rewarding signals, while PPL2 projects to areas important for sensory input and arousal. Flies with disrupted TH function exhibit progressive locomotor deficits that worsen with age^51,52^, paralleling aspects of Parkinsonian motor impairment. However, as in *C. elegans*, Drosophila naturally lacks the α-syn gene and thus requires transgenic expression of hα-syn to model PD–related pathology.

ATP13A2 is represented in Drosophila by a single orthologue (CG32000), and its neuronal depletion consistently points to lysosomal dysfunction as a central pathological event. Neuron-specific knockdown using the elav-Gal4 driver^53^ leads to early accumulation of inclusion body-like protein aggregates as observed by TEM analysis in the brains of 3-day-old flies. These aggregates grew larger in 30-day-old flies, along with an accumulation of Triton-X-100–insoluble ubiquitinated proteins. These proteostasis defects were accompanied by lysosomal accumulation and reduced active form of the lysosomal hydrolase catB. Functionally, these pathological changes were accompanied by impaired climbing ability in the flies^34^.

ATP13A2 loss was combined with α-syn-associated stress to explore how lysosomal vulnerability interacts with proteotoxic challenge. When ATP13A2 silencing was broadly induced using the Glass Multiple Reporter (GMR) promoter^54,55^, overexpression of either WT or A53T and A30P mutant forms of hα-syn^56,57^ resulted in increased Triton X-100–insoluble hα-syn-positive aggregates specifically in flies expressing the A53T variant. In contrast, ATP13A2 silencing driven by Dopa decarboxylase^58^ caused a significant (~50%) reduction in the number of tyrosine hydroxylase (TH)–positive neurons, restricted to the PPL2 cluster. Moreover, flies lacking *ATP13A2* exhibited pronounced disruptions in circadian rhythmicity, reflected by sleep-wake patterns, and the behavioral abnormalities were further exacerbated in flies expressing hα-syn.

Recently, *ATP13A2* emerged as the most relevant hit in a genetic screen aimed at identifying lysosomal genes whose mutation may modify PD penetrance in the context of heterozygous *GBA1* variants. *GBA1*, which encodes the lysosomal enzyme glucocerebrosidase (GCase), represents the most common genetic risk factor for PD^59^. Gu and colleagues showed that heterozygous neuronal *ATP13A2* mutations, together with glial heterozygous *GBA1* mutations, synergistically disrupt lysosomal pH and neuron–glial lipid homeostasis, ultimately triggering neurodegeneration^60^. This is particularly compelling considering earlier evidence linking ATP13A2 to lipid dyshomeostasis^35^, pointing to a convergence of lysosomal pathways whose interaction remains largely unresolved. This convergence gains further relevance from the identification of patients carrying variants in both *ATP13A2* and *GBA1*^60^.

Similar to evidence obtained in *C. elegans*, findings from *D. melanogaster* also point to a conserved role for ATP13A2 in lysosomal homeostasis, potentially indicating that lysosomal dysfunction is a primary driver of the subsequent neurodegenerative process. These studies also support a link between ATP13A2 dysfunction and α-syn aggregation. However, a key limitation is that α-syn expression in these models is not endogenous, thereby lacking physiological relevance. ATP13A2 seems to sensitize neurons to α-syn-related stress rather than acting solely through an α-syn-dependent mechanism.

### Zebrafish and Medaka Fish models

Zebrafish models converge on a central role for ATP13A2 in central nervous system maintenance and lysosomal function, with consequences that extend beyond classical dopaminergic degeneration. Zebrafish represent a valuable model for studying neurodegenerative and movement disorders^61,62^ as the dopaminergic system in zebrafish is functionally conserved, lacking two anatomical structures of the mammalian midbrain dopaminergic system, the SN and the ventral tegmental area^63^. The largest group of zebrafish dopaminergic neurons is primarily located in clusters in the posterior tuberculum (diencephalon)^64^, projecting to the telencephalon, serving as a functional homolog to the mammalian striatum. A vast proportion of them, instead, project to the spinal cord, functioning as the homolog to the mammalian A11 dopaminergic group^65^.

Neuronal populations affected by ATP13A2 deficiency in zebrafish are not restricted to dopaminergic neurons. Transient knockdown models obtained through morpholino-mediated knockdown of zebrafish *ATP13A2* homolog (sharing 50% of homology and 69% of similarity^66^), revealed cerebellar atrophy and defects in motor neurons extension from the notochord^11^. These phenotypes were rescued by co-injecting embryos with wild-type human ATP13A2, contrary to those co-injected with a disease-associated variant linked to ALS^11^. However, a subsequent study published in 2019^67^ showed that the morpholinos used by Spataro and colleagues^11^ exerted ATP13A2-independent effects, which were not addressed in the original work. This observation extends the spectrum of pathologies associated with defects in ATP13A2. Despite limitations, the observed vulnerability of motor neurons suggests that ATP13A2 deficiency may affect multiple neuronal subtypes and not only dopaminergic neurons. This is especially relevant in the case of ALS but also in the case of complicated spastic paraplegia driven by ATP13A2^9,10^. Overcoming limitations of transient knockdown, ENU-induced *ATP13A2*^sa18624^ stable mutant provided evidence linking ATP13A2 to metal homeostasis^67^. Consistent with previous reports of altered metal balance in ATP13A2 deficiency^2^, homozygous mutant zebrafish showed higher sensitivity to Mn^2+^ treatment. Moreover, larvae exposed to a MnCl_2_ treatment displayed significantly enhanced pericardial oedemas, movement loss, and spinal curvature, in addition to the underdevelopment of the swimming, as well as extensive apoptosis in the central nervous system, compared to the WT controls.

Lysosomal maintenance also remains a key aspect in the zebrafish model, as 4 months post fertilization CRISPR/Cas9 *ATP13A2*-KO zebrafish brains showed a significant reduction in the number of dopaminergic neurons in the posterior tuberculum, as well as a significant reduction in the expression of catD^68^. Transmission electron microscopy analysis on 37 months post-fertilization fixed zebrafish brains revealed the accumulation of vesicular structures in lysosomes. Collectively, studies in worms, flies, and zebrafish consistently implicate lysosomal dysfunction as a conserved pathogenic mechanism driving neurodegeneration. This suggests that ATP13A2-related impaired lysosomal homeostasis may be sufficient to promote neuronal loss even in the absence of overt synucleinopathy in these non-mammalian models. Autophagy is not the only mechanism affected by the loss of ATP13A2 function, as shown by proteomic analysis in *ATP13A2*-KO zebrafish brains, revealing putative impairments in intracellular trafficking and a reduction in proteins associated with Golgi apparatus vesicle fusion^68^.

In parallel, ATP13A2 deficiency has also been modeled in medaka fish (*Oryzias latipes*). Like zebrafish, medaka fish have small, transparent embryos. However, medaka fish are significantly less used in the field of biomedical research. Yet, there are valid reasons why one may choose medaka fish over zebrafish: the medaka fish genome is significantly smaller than the zebrafish one^69^, and they have a clear XX/XY sex determination system^70^, while sex in zebrafish is polygenic and temperature dependent. On the other hand, medaka fish lack the regenerative capacity that makes zebrafish so valuable in the field^71^, and consequently, less infrastructures are available for medaka fish. The amino-acid sequence of medaka fish ATP13A2 shows 51.3% homology to the human ATP13A2 protein^39^. Despite normal gross development, ATP13A2 homolog-deficient medaka obtained with the Targeting Induced Local Lesions in Genomes (TILLING) strategy, displayed a reduced lifespan^72^. Age-dependent loss of TH⁺ neurons was mild; however, this mutant fish also displayed noradrenergic neuronal loss. A marked decrease in the lysosomal enzyme catD activity was reported, while catK and catL levels remained unchanged. Notably, the relatively modest dopaminergic neuronal loss was not associated with overt locomotor impairment, suggesting that partial neuronal degeneration may be insufficient to elicit clear motor phenotypes in this model.

Taken together, zebrafish and medaka models consistently implicate ATP13A2 in lysosomal homeostasis, dopaminergic neuron maintenance, and protection against metal-induced toxicity. However, important limitations remain, including the extent to which the neuronal loss translates into motor dysfunction, if any, and why these pathological features are detectable only in late zebrafish adulthood, failing to parallel the KRS juvenile-onset. Interestingly, however, zebrafish suggest that ATP13A2 may also play a key role in the maintenance of diverse neuronal populations, such as motor neurons, an aspect that remains largely unexplored.

## Mammalian models

### In vitro models

Cellular models are a pivotal tool for basic pathophysiological research as well as drug development and compound testing in neurodegenerative diseases. Over the last decade, ATP13A2 research has generated a wide array of in vitro models, with the field rapidly advancing toward increasingly sophisticated and technically challenging experimental systems. Human immortalized cell lines are easy to use and highly reproducible, but often lack physiological relevance. Primary cultures better reflect in vivo biology, yet are limited by lifespan, variability, and accessibility. Patient-derived fibroblasts preserve genetic background but have restricted relevance and proliferation. iPSCs offer renewable, patient-specific, multi-lineage models, although they are technically demanding and not fully mature. Despite their limitations, all models provide valuable insights into most mechanistic aspects of ATP13A2 function, consistently indicating that lysosomal dysfunction is a central consequence of ATP13A2 loss.

Across immortalized cell lines, primary cultures, and patient-derived cells (fibroblasts or induced pluripotent stem cells), ATP13A2 deficiency converges on impaired lysosomal degradation, impaired metals and polyamines handling, defective autophagy, and secondary mitochondrial dysfunction.

While overexpression of ATP13A2 in immortalized cell lines confers protection against metal-induced toxicity^73^, the overexpression of an inactive variant displays a significant sensitivity to ferric chloride exposure, rescued by the expression of native human ATP13A2 protein^37^. Immortalized neuronal cell lines (BE(2)-M17^30^ and SH-SY5Y^39^) with *ATP13A2* silencing show compromised lysosomal fitness, leading to an increased number of lysosomes with reduced degradative capacity. Lysosomal abnormalities were accompanied by disrupted autophagic flux, as knockdown cells showed resistance to autophagy induction. This disruption is tightly linked to mitochondrial stress: defective autophagy results in mitochondrial accumulation, elevated oxygen consumption, and increased ROS production^33,39^. However, ATP synthesis remains largely unaffected. More recently, defective polyamine transport^26^ has emerged as a key mechanistic link between ATP13A2 loss and mitochondrial oxidative stress. Polyamines, which act as potent intracellular antioxidants, are redistributed to the mitochondria where they locally counteract ROS^33^.

Similar pathogenic features are recapitulated in primary neuronal cultures, which, despite their limited lifespan, more closely reflect physiological neuronal biology and reinforce the relevance of these findings in a more native cellular context. Mouse primary neurons with *ATP13A2* silencing show mitochondrial accumulation^39^. These observations corroborate the link between lysosomal dysfunction and polyamine dyshomeostasis, suggesting that impaired polyamine export contributes to neuronal vulnerability alongside the defects in autophagy, mitochondrial turnover, and metal handling observed in ATP13A2-deficient cells. In rat primary neurons, *ATP13A2* silencing not only impairs neurite growth but also promotes mitochondrial fragmentation and enhances sensitivity to metal ions, highlighting the vulnerability of these cells to bioenergetic and oxidative stress^74^.

Supporting the importance of ATP13A2 in PD pathophysiology, post-mortem nigral tissues from sporadic PD patients display a significantly reduced ATP13A2 expression via immunohistochemical analysis^30^. Fibroblasts and induced pluripotent stem cells (iPSCs) further reveal how these cellular defects manifest in patient-derived cells. Fibroblasts from PD, KRS, and HSP patients harboring *ATP13A2* point mutations exhibit a dramatic increase in lysosomal number^11,30,46^. Electron microscopy revealed more numerous autolysosomes containing undigested material, alongside impaired catD maturation, compromised lysosomal pH, and reduced catD activity^30^. This decreased proteolysis is restored by overexpression of WT ATP13A2^30,46^. Alongside lysosomal defects, a significant increase in ubiquitinated proteins is observed^32^. Severe mitochondrial impairments, including significantly reduced membrane potential and ATP synthesis and elevated mitochondrial mass, were observed in KRS and HSP patient-derived fibroblasts^11,75^ Human olfactory neurospheres from a heterozygous patient with a mutation in the *ATP13A2* gene were more sensitive to Zn²⁺ challenge, with synchrotron X-ray fluorescence revealing elevated intracellular Zn²⁺ accumulation, but not Mn²⁺ ^76^.

Patient-derived iPSCs have only recently been generated and remain relatively underexplored^77,78^. Tsunemi and colleagues first studied human iPSCs from two male KRS patients differentiated into neurons and astrocytes. This ATP13A2-deficient cell model results in severe impairment of endocytosis and proteolysis, as evidenced by reduced epidermal growth factor receptor internalization and degradation rates^78^. The ATP13A2 loss-of-function impaired astrocyte internalization, in turn elevating neuronal α-syn levels and suggesting a protective role against propagation^78^. A second study showed that ATP13A2 deficiency reduces extracellular vesicle numbers from neurons and astrocytes but increases α-syn PFFs per vesicle^77^. Lately, siRNA *ATP13A2* iPSC-derived neurons, and *ATP13A2* KO cell lines exhibited a lysosomal polyamine accumulation leading to the inhibition of lysosomal GCase^36^. These findings point to a previously underappreciated link between ATP13A2 function and lipid homeostasis, which was partially suggested in immortalized cells^35^. Notably, the work by Marcos and colleagues focused on ATP13A2 overexpression, whereas more recent evidence associates lipid dyshomeostasis with ATP13A2 loss-of-function, highlighting the need for further mechanistic characterization of this pathway and of the interaction with GBA1.

Across immortalized lines, primary neurons, patient fibroblasts, and iPSC-derived cells, these findings converge to a rather unified picture: ATP13A2 maintains lysosomal function and polyamine homeostasis, and its loss triggers a cascade of defects including impaired autophagy, mitochondrial stress, metal and polyamine dyshomeostasis, and compromised handling. More recently, in vitro models, along with evidence in *Drosophila*^60^ and in mice^36^, point also to an alternative pathway that links lysosomal dysfunction to impaired lipid homeostasis. While immortalized cells allow reproducible mechanistic dissection, patient-derived cells are essential to capture mutation-specific effects and genetic background-dependent variability, emphasizing the translational relevance of these models despite technical and mutation challenges. Studies in patient-derived fibroblasts or iPSCs remain a priority when feasible, as they enable investigation of single-point mutation effects, whereas most studies examined in this review rely on complete protein deletion.

### Mouse models

Rodent models remain an indispensable platform and have significantly contributed to our understanding of ATP13A2-linked neurodegeneration over the years. Across multiple genetic strategies, a consistent picture has emerged in which ATP13A2 loss primarily compromises lysosomal homeostasis and glial function, while producing only mild and late-onset motor phenotypes.

Interestingly, and divergent with clinical observations, constitutive *ATP13A2*-deficient mouse models exhibit subtle motor alterations that emerge late in life. Motor symptoms include impaired beam crossing, reduced spontaneous activity, altered hindlimb stepping or clasping during tail suspension, and gait abnormalities at a late age, and often with a male bias^79^^,^^80^. Moreover, a brain-specific *ATP13A2* KO generated using the loxP model and Nestin-Cre driver system also developed mild motor deficits from 18 months of age, including reduced fall latency both in accelerating rotarod and in wire hang tests, with no impairments observed at earlier time points^81^. Despite these relatively mild behavioral phenotypes, several ATP13A2 mouse models display pronounced glial alterations, suggesting that neuroinflammatory processes may represent an early and prominent feature of ATP13A2 deficiency. Brain sections from germinal *ATP13A2*-deficient mice revealed widespread GFAP and Iba1-positive gliosis across multiple regions. Astrocytes appear to be particularly affected by the absence of ATP13A2, as astrogliosis was observed broadly in several brain areas^80,82^, including the cortex, striatum, hippocampus, cerebellum, thalamus, and midbrain^80^. In the *ATP13A2*-floxed KO mice presented by Scultheis and colleagues, astrocytes exhibit an exacerbated inflammatory response upon MPP⁺ treatment, characterized by increased cytokine release and activation of the NLRP3 inflammasome^83^. While germline KO mice exhibit progressively increasing astrogliosis, the AAV-Cre conditional model showed elevated GFAP, which is largely attenuated by 10 months, suggesting that astrocyte activation may be a transient response^82^.

Despite the mild decrease in motor performance and the presence of neuroinflammation, the integrity of the nigrostriatal pathway is not affected by the germline ATP13A2 loss-of-function, even in aged mice^79,80,84^. However, the only robust loss of TH-positive neurons is observed with the adult-onset *ATP13A2*-floxed KO mice, which induces a ~35% TH-positive neuronal loss^82^, further supported by the adult-onset silencing of *ATP13A2* in a non-human primate model^85^.

Nevertheless, the dysfunction of the autophagy process, with the accumulation of lipofuscin, p62, LC3, and insoluble ubiquitinated proteins is a shared mechanism between ATP13A2 mouse models. Indeed, pathological features include an accelerated accumulation of autofluorescent lipofuscin-like deposits in the cerebellum and hippocampus^79^. Aged KO mice showed approximately a 2-fold increase in lipofuscin in the cortex in comparison to WT mice^80^. Ultrastructural analyses revealed electron-dense lamellated inclusions in the neurons of the SN, indicative of lysosomal storage pathology^82^. Additionally, defects in lysosomal enzyme processing were observed, including impaired maturation of catD and the aberrant accumulation of subunit c of mitochondrial ATP synthase, a hallmark of disrupted lysosomal degradation pathways^81^. LAMP2 and p62 positive inclusions are also increased in the conditional adult-onset *ATP13A2*-KO model, but these alterations only became apparent at 10 months post-injection and were not detectable at earlier time points^82^. The disconnection between the autophagy-related pathology and the dopaminergic integrity represents a key limitation of germline mouse models, as it contrasts with the early and severe clinical presentation observed in patients.

Notably, no consistent differences in pathological α-syn aggregation were detected across the different models. However, increased levels of Triton X-100-insoluble α-syn were reported in the hippocampus of *ATP13A2*-null mice^79^, and these levels were further increased following manganese treatment^86^. *ATP13A2*-null mice were further used to investigate the role of ATP13A2 in hα-syn propagation by injecting hα-syn preformed fibrils (PFFs) in the striatum. Despite no differences in α-syn propagation between WT and *ATP13A2* KO mice three months post-injection^77^, ATP13A2 loss was associated with a marked reduction in brain-derived extracellular vesicles, suggesting altered vesicle release or homeostasis. This finding aligns with proteomic data by Nyuzuki and colleagues in the CRISPR-mediated *ATP13A2* KO zebrafish^68^. Injection of α-syn PFFs into the striatum did not exacerbate α-syn pathology and TH-positive neuronal loss in a subsequent study^84^, suggesting that ATP13A2 deletion does not increase the vulnerability of dopaminergic neurons to the neurotoxic effects of α-syn PFFs in mice^84^.

Recent work using the mouse model developed by Schultheis and colleagues^79^ examined whether dysregulated ATP13A2 activity leads to polyamine dyshomeostasis in the brain. Metabolomic and lipidomic profiling of 5- and 15-month-old *ATP13A2* KO mice revealed an increase in polyamines, including spermine and N1-acetylspermine in the cortex, and N1-acetylspermine in the hippocampus. In parallel, the study identified a sustained increase of lipid hydrolysis–related metabolites in both regions, particularly those linked to the GCase. These results suggest that loss of ATP13A2 function may disrupt GCase activity, pointing to a broader impairment of lysosomal lipid metabolism^36^. Importantly, the connection between ATP13A2 function and lipid homeostasis remains poorly explored, particularly considering the emerging interplay between two proteins implicated in genetic forms of PD, ATP13A2 and GCase.

Concerning partial *ATP13A2* deletion, significant lipofuscin accumulation, microgliosis, and astrogliosis were also observed in heterozygous mice, along with cognitive impairments, polyamine dyshomeostasis, and markedly elevated α-syn pathology compared with KO animals. Total α-syn and phosphorylated α-syn levels were analyzed across four brain regions (prefrontal cortex, striatum, ventral midbrain, and cerebellum) at 3, 12, and 18 months of age. Heterozygous mice consistently showed the most pronounced alterations, with significantly increased α-syn levels compared with both WT and KO animals at 3 and 18 months, whereas at 12 months, the increase was restricted to the cerebellum. A similar pattern was observed for phosphorylated α-syn^87^.

Mouse models highlight that ATP13A2 loss primarily disrupts lysosomal homeostasis, polyamine and astrocyte function, creating a permissive environment for neurodegeneration. Overt dopaminergic neuron loss requires either adult-onset, region-specific deletion or additional stressors, emphasizing that ATP13A2-linked neurodegeneration is context-dependent and unfolds over time. Both germline and conditional models reveal lysosomal abnormalities and polyamine/lipid dyshomeostasis, confirming that lysosomal dysfunction is a core consequence of ATP13A2 loss. Across models, astrocytes consistently show exaggerated inflammatory responses. Despite low ATP13A2 expression, astrocytes seem to play a central, underappreciated role in disease progression.

### Rat models

To our knowledge, two articles report the use of an ATP13A2-related rat model. First, in 2015, Daniel and colleagues described an AAV-mediated vector injection that permitted the overexpression of human ATP13A2, or the expression of an ATPase-deficient form of ATP13A2 (D513N) in Sprague-Dawley rats^88^. This article demonstrated that viral co-overexpression of the human form of ATP13A2 and α-syn in the SN was insufficient to induce neuronal loss and dopaminergic depletion. Moreover, the level of human α-syn in the striatum remained unchanged, as did motor parameters, after injection of AAV-hATP13A2. However, the expression of the D513N ATP13A2 mutated form was sufficient to induce dopaminergic (TH-positive) and neuronal (Nissl-positive) degeneration in the SN, 14 weeks post-injection of the AAV vector in the SN of Sprague-Dawley rats, with the apparition of motor asymmetry deficit assessed by amphetamine-induced rotation test at 8- and 12-weeks post AAV-injection^88^.

Recently, a full *ATP13A2* KO rat model was generated using CRISPR-Cas9 to remove the *ATP13A2* sequence between exon 4 and exon 6, leading to a frameshift and a premature termination codon in exon 7^89^. This deletion induces the abolition of *ATP13A2* mRNA expression. Although this *ATP13A2* KO is not embryonically lethal, it implies multiple neurodevelopmental delays, such as the eye-opening reflex, startle response, or negative geotaxis reflex. Moreover, the authors demonstrated a critical period for motor development dependent on ATP13A2 between the 9th and the 12th day of life in *ATP13A2* KO pups through a spontaneous locomotion test^89^. Interestingly, this model shows a fine motor skill deficit at 12 months in the single-pellet reaching task, consistent with KRS Parkinsonism symptoms, and a hyperactive phenotype already observed in a KRS patient^4^. However, as shown in multiple *ATP13A2* KO animal models, this rat does not display any nigrostriatal neurodegeneration, even at a late age (16 months old), in either the SN or the striatum. Nevertheless, electrophysiological alterations in ATP13A2-deficient rats are observed in dopaminergic neurons of the SN at 3 and 6 months old. Measurements were conducted with a glass micropipette in isoflurane-anesthetized animals, which allows the characterization of a significant increase in the burstiness and a reduction in the action potential amplitude. These electrophysiological results could help to interpret the observed phenotype in *ATP13A2* KO rodents. This type of experiment should be conducted in other models to gather information on the ATP13A2 role in the dopaminergic neurons’ electrophysiological balance.

Additionally, ATP13A2-deficient rats demonstrated a decrease in autophagosome vacuoles and the accumulation of early- and late-autolysosomes in the dopaminergic neurons of the SN by electron microscopy. This indicates that autophagic flux is altered in this rat model, consistent with results obtained from the diverse ATP13A2-related models. This ALP dysfunction was further supported by the use of a viral vector expressing a pH-sensitive tandem fluorophore coupled to the LC3 protein, showing an increased ratio between the number of autolysosome-related vesicles and autophagosomes in ATP13A2-deficient animals. Moreover, this study also assessed the impact of viral-mediated overexpression of human A53T-mutated α-syn and human tyrosinase in *ATP13A2* KO rats, demonstrating phosphorylated α-syn accumulation and aggregation, which was not observed in the *ATP13A2* KO-only rats.

The ATP13A2-deficient rat model, therefore, appears to be an interesting alternative to the mouse model in the study of ALP dysfunction and the impact of the ATP13A2 deletion on the onset of motor symptoms in germline KO-generated models. This animal model recapitulates the major finding described in *ATP13A2* KO mice models, with important alteration of ALP flux without dopaminergic neurodegeneration, but while demonstrating electrophysiological impairment, heavy metal dyshomeostasis, and inflammatory process induced by the loss of ATP13A2 function. It therefore seems worthwhile to characterize the differences between this *ATP13A2* KO rat and a possible adult-onset induced deletion of *ATP13A2* in the rat model to better apprehend the compensatory mechanisms involved at germline induction.

### Canine models

Among the various species susceptible to developing neuronal ceroid lipofuscinosis (NCL)^90^, two dog breeds have been identified as potentially spontaneously carrying *ATP13A2* mutations related to NCL. First, in 2011, Farias and colleagues described a homozygous truncating mutation in *ATP13A2* in Tibetan terriers, responsible for adult-onset NCL^91^. This study used a database of isolated DNA from 439 Tibetan terriers^92^ and 36 Tibetan terriers with a presumptive diagnosis of NCL. Furthermore, Wöhlke and colleagues also used samples from 24 NCL-affected Tibetan terriers and 1347 unaffected Tibetan terriers^93^. Affected dogs presented a single base-deletion in the exon 16, *ATP13A2:c.1623delG*^91^ or *ATP13A2:c.1620delG*^93^, leading to a frameshift mutation and a premature termination codon. This shortens the ATP13A2 protein by 69 amino acids, impacting the P-type cation-transporting ATPase superfamily and sodium/potassium-transporting ATPase signature, as well as the E1-E2 ATPases phosphorylation site and the haloacid dehalogenase-like hydrolase site^93^. Regarding the phenotype, affected dogs show behavioral changes reported by their owners around 6 years old, such as enhanced aggressiveness, disorientation, cognitive and learning decline, and cerebellar ataxia. Some symptoms, such as cerebellar ataxia, differ from what is observed in KRS, with the absence of Parkinsonism or myoclonus in the Tibetan terrier. Indeed, SN and other basal ganglia structures seem relatively more resistant to ATP13A2 deficiency than in humans. Nevertheless, magnetic resonance imaging observations revealed diffuse and significant brain atrophy with dilated ventricles, a feature observed in KRS patients depending on disease duration^13,94^. They correlated with a hypointense putaminal signal on DaTSCAN single-photon emission computed tomography^95^, but no alterations were observed in the caudate nuclei. Also, lipofuscin-like autofluorescent elements are observed in the retina, the optic nerve, the cerebellar cortex, the molecular, Purkinje, and granular layers of the cerebellum of *ATP13A2*-mutated dogs^91,93^.

More recently, Australian Cattle Dogs have been screened to possibly carry the *ATP13A2* mutation, leading to loss of function and NCL. Indeed, a female and two of her offspring were identified with a homozygous c.1118 C > T variant in ATP13A2, predicting a nonconservative p.(T373I) substitution in the E1-E2 ATPase domain^96^. Affected Australian Cattle dogs present a similar phenotype compared to ATP13A2-deficient Tibetan terriers, as well as the presence of lipofuscin-like elements in the molecular and Purkinje cell layer, the meninges, and LAMP1-positive large neurons in the deep cerebellar nuclei, suggesting a lysosomal-derived origin. Furthermore, astrogliosis in the deep cerebellar nuclei and in the cerebral cortex has been shown in this breed with glial fibrillary acid protein (GFAP) staining, a feature observed, for example, in the *ATP13A2* KO rat model^89^. Canine models are the only ones known, apart from humans, to develop spontaneous *ATP13A2*-pathological mutation; they give interesting insights into ATP13A2 loss of function not artificially induced in the pathological process, with many symptoms and significant anatomopathological hallmarks observed in NCL and KRS-affected patients.

### Non-human primate model

To date, only one ATP13A2-related non-human primate (NHP) model has been described in the literature. In a pilot study published in 2024, Sikora and colleagues injected a lentiviral vector expressing an *ATP13A2* shRNA targeting human *ATP13A2* with the U6 promoter, previously validated by Dehay and colleagues^30^, into the SN of two adult female macaques^85^. After five months, ATP13A2 immunofluorescence staining revealed a decreased level in dopaminergic SN neurons, confirming the efficiency of the shRNA previously described in vitro^30^. The adult-onset induction of the *ATP13A2* silencing resulted in a significant ~28% TH-positive cell loss in the SN, but also a clear loss in the putamen and the caudate nucleus, supporting the idea of a compensatory mechanism for dopaminergic resilience installed at youth for ATP13A2 loss of function, but not at adult-onset^85^. Indeed, an article by Erb and colleagues, published jointly, describes that adult-onset deletion of ATP13A2 in mice also leads to a progressive nigrostriatal pathway dopaminergic degeneration^82^. In a commentary on these two articles, Veerle Baekelandt highlights the importance of pathological induction in adulthood to mimic the KRS phenotype and suggests some clues as to the processes involved, such as neuroinflammation as a secondary trigger or a reduction in compensatory mechanisms at a late age^97^. The loss of function of ATP13A2 in this NHP model displays a synucleinopathy hallmark: the presence of phosphorylated α-syn in the SN. At an autophagic level, the accumulation of LC3- and LAMP2-positive vacuoles, respectively referring to autophagosome and lysosome-like vacuoles, in dopaminergic neurons confirms an ALP dysfunction. Moreover, ATP13A2 deficiency in the SN leads to dysregulation of heavy metal homeostasis in the region, measured by synchrotron radiation X-ray fluorescence, with a significant increase in iron and manganese levels and a significant decrease in copper levels. These dysregulations are correlated with clinical observations in KRS patients, especially for iron accumulation in the basal ganglia^98,99^. Thus, this first study of ATP13A2-related NHP using shRNA in adult animals induces significant dopaminergic depletion, mimicking anatomopathological observation of KRS patients, and proves its usefulness for the future of ATP13A2-related modelling. Even if the reported results are significant in the development and comprehension of KRS, it is important to underline that the study has been conducted on only two animals. Further investigation will permit a better comprehension of the ATP13A2 role in the neurodegeneration process with a larger cohort and its impact on behavior and motor functions in NHP.

### What we have learned

A striking aspect of progress in modeling ATP13A2-linked neurodegeneration is the wide range of animal species used, from invertebrates to mammals. This diversity has been advantageous, as different models capture distinct aspects of ATP13A2 biology and pathology depending on species, genetic background, and experimental approach. Importantly, current ATP13A2 models are not faithful models of specific clinical entities such as PD, KRS, NCL, HSP, or ALS; rather, each reproduces only selected features of a complex and still poorly understood disease spectrum.

Nevertheless, key insights have emerged. ATP13A2 encodes a P5-type lysosomal ATPase essential for lysosomal integrity, ion and polyamine homeostasis, and crosstalk with mitochondria and the ER. Loss of ATP13A2 function disrupts lysosomal acidification and autophagy, increases metal sensitivity, promotes α-syn aggregation, induces mitochondrial dysfunction and oxidative stress, and impairs degradation of damaged proteins and organelles. Across models, these defects converge on lysosomal failure as a central pathogenic hub.

Evidence suggests a pathogenic feedback loop in which ATP13A2 deficiency impairs lysosomal clearance of α-syn, leading to its accumulation and further lysosomal damage, like mechanisms described for GBA. Lysosomal dysfunction may also increase susceptibility to lysosomal membrane permeabilization, promoting mitochondrial damage and cell death. These processes likely act synergistically in an age-dependent manner, ultimately contributing to dopaminergic neuron loss.

Advanced in vivo models have refined these insights: while germline KO mice show limited nigrostriatal degeneration, adult-onset conditional deletion in mice and targeted silencing in NHP induce robust Parkinson-like pathology, more closely mirroring human disease progression. Overall, each model represents only one piece of a complex puzzle, underscoring the need to test therapeutic strategies across multiple models and mechanisms.

### Conclusion, gaps challenges and perspectives

Substantial progress in our understanding of the molecular function of ATP13A2 has been made over the last five years. However, despite the availability of many different animal models, each recapitulating certain key aspects of ATP13A2-driven pathology, they are ultimately characterized by severe limitations that prevent translational progress. KO rodents, for example, fail to recapitulate critical pathological aspects characteristic of human KRS, such as progressive neuronal loss and strongly debilitating motor disorders. These differences in end-stage pathology suggest the presence of species-specific compensatory mechanisms, rendering them inherently only partially reliable, especially when it comes to designing therapeutic interventions. Nonetheless, ATP13A2-related models share characteristic features of the KRS, such as an important dysfunction of the ALP system, with the accumulation of lysosomal-related vesicles, a deficit in the polyamine and heavy metal homeostasis, and toxic sensitivity. Moreover, inflammation and astrogliosis are recurrent observations in animal models, uncovering an interesting mechanism that remains to be elucidated. From this perspective, improving the characterization of the NHP model by increasing the cohort size and elucidating the compensatory mechanisms in *ATP13A2*-KO rodent pups seems to be an important future step in the comprehension of ATP13A2 roles. Overall, animal models described in this review have been fundamental to understanding numerous aspects of ATP13A2 biology and physiology, thereby elucidating the mechanisms underlying disease pathology. iPSC and other non-animal models may be crucial for the next steps in the comprehension of ATP13A2. However, as efforts increasingly shift toward humanized models and precision medicine approaches, it remains important not to overlook the substantial evidence accumulated over the years in vitro from patient-derived fibroblasts and iPSC-derived models. In many cases, available models are based on the complete knockout of the gene, thereby overlooking the specific molecular and phenotypic effects of disease-causing point mutations. By contrast, patient-derived fibroblasts and, more recently, iPSCs offer superior suitability for capturing these effects. This translational gap remains only partially explored^36,78^ in modeling ATP13A2-linked neurodegeneration. Despite lacking the physiological context that animal models provide, iPSC models capture human genetic background and vulnerabilities that drive disease progression. A successful approach in the future must integrate a complementary pipeline combining iPSC platforms and animal models for the design and optimization of effective therapeutic interventions. At the same time, the analysis of new tissue samples from KRS patients or PD patients harboring ATP13A2 mutations would enable the community to better understand the pathological role of the ATP13A2 loss of function and help to validate phenotypes observed in the different models generated over the last few years. These multiple animal and cellular models, combined with patient-derived samples, will advance knowledge about the ATP13A2 specificity in physiologic and pathologic conditions. Indeed, multiple aspects remain to be clarified, such as the role of ATP13A2 on α-syn homeostasis and its role in glial cells. Although the role and main substrates of ATP13A2 are known, the coupling and stoichiometry between its role as a polyamine exporter^2,34^ and H+/K+ ATPase^32^ remain to be elucidated. Moreover, the conformational states of the mutated forms of ATP13A2 observed under cryo-electron microscopy remain an unexplored realm, possibly interesting to better understand the coupling process and druggable allosteric pockets. In the future, we hope that the ATP13A2-linked animal models exposed in this review will permit the development and the preclinical validation of therapeutic strategies for KRS, but also PD and diseases linked to lysosomal dysfunction.

## Data Availability

No new datasets were generated or deposited.
